# Socioemotional wealth, family governance, and organizational belonging in multigenerational family firms: a social identity perspective

**DOI:** 10.3389/fpsyg.2026.1772135

**Published:** 2026-04-22

**Authors:** Ying Wang, Kang Meng, Yousef Mohammad Karimi

**Affiliations:** 1 Department of Finance and Economics, Xuchang Vocational Technical College, Xuchang, Henan, China; 2 Department of Business Management, Payame Noor University, Tehran, Iran

**Keywords:** family firms, family governance, organizational belonging, social identity, socioemotional wealth

## Abstract

The purpose of this study is to examine the relationship between socioemotional wealth (SEW) and employees’ organizational belonging (OB) in family firms and to investigate the mediating role of family governance (FG) in this relationship. The statistical population of the study consisted of employees working in family businesses, from which a sample was selected using a survey-based approach. Data were collected through a structured questionnaire and the sampling process followed a convenience sampling method among employees of family firms. To analyze the proposed research model, structural equation modeling (SEM) was employed using AMOS software. In addition, the mediating role of family governance was tested using the bootstrapping method with 5,000 resamples and a 95% confidence interval. Multi-group analysis was also conducted to examine differences across demographic groups. The results indicate that socioemotional wealth has a positive and significant effect on both family governance and organizational belonging. Furthermore, family governance has a positive and significant effect on employees’ organizational belonging. The mediation analysis shows that family governance partially mediates the relationship between socioemotional wealth and organizational belonging. The multi-group analysis further reveals that the relationship between family governance and organizational belonging is stronger among female employees and older respondents.

## Introduction

Family firms constitute one of the most dominant forms of business organization worldwide and play a significant role in economic development, employment creation, and long-term organizational sustainability (Salvato et al., 2019; Rehman et al., 2023). These firms are characterized by the overlap of family and business systems, which shapes their governance structures, decision-making processes, and organizational priorities in ways that differ from non-family organizations (Barrett and Moores, 2020; Casprini et al., 2024). Because ownership and management are often influenced by family interests and values, family firms frequently pursue goals that extend beyond purely financial objectives (Kammerlander, 2022; Eddleston and Jennings, 2024).

One of the most influential theoretical perspectives explaining the unique behavior of family firms is socioemotional wealth (SEW), which refers to the non-economic value family owners derive from their involvement in the firm, including family control, identity, emotional attachment, and the intention to preserve the family legacy across generations (Berrone et al., 2012; Kammerlander, 2022; Chen et al., 2024). The SEW perspective argues that family firms often make strategic choices aimed at protecting these socioemotional endowments even when such decisions may involve economic trade-offs (Berrone et al., 2012). As a result, socioemotional wealth has been widely used to explain family firm behavior in areas such as governance, corporate responsibility, and long-term strategic orientation (Rehman et al., 2023; Baltazar et al., 2025).

Although a substantial body of research has examined the influence of socioemotional wealth on strategic decisions and firm-level outcomes, most studies have focused primarily on the perspectives of family owners and top managers (Berrone et al., 2012; Kammerlander, 2022). Consequently, relatively limited attention has been given to how socioemotional priorities influence employees within family firms and shape their perceptions of the organizational environment (Casprini et al., 2024; Lukito et al., 2025). This limitation is important because employees represent a critical stakeholder group whose attitudes and experiences can significantly influence organizational performance and sustainability (Neckebrouck et al., 2018; Klein et al., 2025).

One employee-related outcome that has gained increasing attention in organizational research is the concept of organizational belonging. Organizational belonging refers to an individual’s perception of being accepted, valued, and psychologically connected to the organization and its members (Blau et al., 2023; Urrila et al., 2025). A strong sense of belonging has been associated with numerous positive outcomes, including stronger organizational commitment, higher engagement, and improved collaboration among employees (Bryer, 2020; Davis et al., 2022). Conversely, a lack of belonging can lead to reduced organizational attachment and lower levels of employee well-being (Kim et al., 2019; Stratton-Maher et al., 2025). Because family firms often emphasize relational values, identity, and long-term relationships, the concept of belonging may be particularly relevant in these organizational contexts (Eddleston and Jennings, 2024).

A key mechanism through which socioemotional priorities may influence employee experiences is family governance. Family governance refers to the set of formal and informal structures that regulate the relationship between the family and the business, including mechanisms such as family councils, family meetings, governance policies, and communication frameworks (Berent-Braun and Uhlaner, 2012; Ginesti et al., 2023). These governance mechanisms are designed to coordinate family involvement in the firm, clarify roles and expectations, and support effective decision-making processes (Gupta and Chauhan, 2023; Tawfik et al., 2022). Effective governance structures can also enhance transparency and legitimacy within organizations, thereby shaping how employees perceive fairness, stability, and organizational coherence (Honey et al., 2025; Lv et al., 2025).

Prior research suggests that governance arrangements can play a critical role in translating family values into organizational practices and behaviors (Gupta and Chauhan, 2023; Ginesti et al., 2023). However, empirical research has rarely examined whether family governance functions as a mediating mechanism linking socioemotional wealth to employee-level outcomes such as organizational belonging. Understanding this relationship is important because governance structures may represent the institutional channel through which family-centered priorities become visible and meaningful to employees (Berent-Braun and Uhlaner, 2012; Casprini et al., 2024). Consequently, a clearer understanding of the governance mechanisms through which SEW influences employees remains an important gap in the family business literature.

Furthermore, employee perceptions of governance structures may vary across demographic characteristics. Differences in gender and age, for example, may influence how individuals interpret organizational procedures, relational dynamics, and fairness within the workplace (Karami et al., 2024; Behm-Morawitz and Villamil, 2019). Investigating such differences can provide a more nuanced understanding of how governance mechanisms influence employees’ sense of belonging in family firms. In light of these considerations, the present study aims to examine the relationships among socioemotional wealth, family governance, and employees’ organizational belonging in family firms. Specifically, the study investigates whether family governance serves as a mediating mechanism through which socioemotional priorities influence employees’ sense of belonging within the organization. Accordingly, the study addresses the following research questions:

*RQ1*: Does socioemotional wealth influence employees’ organizational belonging in family firms?

*RQ2*: Does socioemotional wealth influence the development of family governance mechanisms in family firms?

*RQ3*: Does family governance influence employees’ organizational belonging?

*RQ4*: Does family governance mediate the relationship between socioemotional wealth and employees’ organizational belonging?

*RQ5*: Do the relationships among socioemotional wealth, family governance, and organizational belonging differ across demographic groups such as gender and age?

## Literature review

### Organizational belonging in family firms

Organizational belonging refers to individuals’ subjective experience of feeling emotionally connected to, accepted by, and included within an organization (Baumeister and Leary, 1995; Jena and Pradhan, 2018). In the context of family firms, organizational belonging reflects the extent to which family members feel psychologically attached to the family-owned organization as a meaningful social space, beyond their formal roles or ownership positions (Nadine, 2022; Blau et al., 2023). Prior research suggests that a stronger sense of belonging is associated with positive organizational experiences such as higher engagement, loyalty, and willingness to contribute beyond contractual obligations (Chatzidakis and Shaw, 2018; Song and Kim, 2021; D’Angelo et al., 2023). Despite its relevance, organizational belonging has received comparatively limited attention in family business research, particularly when compared to constructs such as organizational identification or commitment.

Drawing on a social identity perspective (Ashforth and Mael, 1989), organizational belonging can be understood as an affective and experiential state that emerges when individuals perceive themselves as accepted members of a valued organizational in-group. While social identity theory emphasizes the cognitive process through which individuals define themselves in terms of group membership, organizational belonging captures the emotional and relational dimension of this experience namely, how individuals feel within the organizational community. In family firms, where organizational boundaries often overlap with family ties, these emotional experiences may be especially salient yet also more fragile, particularly across generations (Behm-Morawitz and Villamil, 2019; Karayianni et al., 2023).

It is important to distinguish organizational belonging from closely related constructs that are frequently used in the family business literature. Organizational identification refers to the degree to which individuals cognitively define themselves in terms of the organization and internalize its values and identity (Ashforth and Mael, 1989; Berrone et al., 2012). Identification is primarily a cognitive and value-based process linked to self-definition and alignment with organizational goals. In contrast, organizational belonging emphasizes affective experiences of inclusion, acceptance, and interpersonal support within the organizational context (Baumeister and Leary, 1995). A family member may strongly identify with the family firm’s legacy or values, yet still experience low organizational belonging if everyday interactions are characterized by exclusion, conflict, or perceived unfairness.

This distinction is particularly relevant in multigenerational family firms. As ownership structures become more complex and family involvement diversifies across generations, emotional attachment to the organization cannot be assumed to persist automatically (Baltazar et al., 2025). Differences in generational expectations, roles, and decision-making authority may weaken feelings of inclusion, even among family members who continue to identify strongly with the family firm. Accordingly, organizational belonging represents a distinct and theoretically meaningful outcome that captures how family members experience their place within the organizational community.

Within the socioemotional wealth (SEW) framework, organizational belonging relates to the family’s desire to preserve non-economic utilities such as emotional attachment, family identity, and social ties within the firm (Berrone et al., 2012). However, organizational belonging is not treated here as synonymous with socioemotional wealth. Rather, it is conceptualized as an individual-level psychological experience that may co-vary with broader family-level priorities emphasized by SEW, while remaining analytically distinct from them. Similarly, organizational belonging is differentiated from family social capital and governance practices. Whereas social capital refers to relational resources embedded in family ties, and governance practices denote institutional arrangements guiding interaction and decision-making, organizational belonging captures how individuals emotionally experience these relational and structural contexts.

In this study, organizational belonging is therefore conceptualized as an affective, individual-level construct reflecting family members’ feelings of connection, acceptance, and inclusion within the family firm. By focusing on organizational belonging, the study responds to calls for greater attention to the emotional and experiential dimensions of family involvement in business and provides a theoretically grounded outcome variable that aligns with both socioemotional wealth logic and a social identity perspective.

### Socioemotional wealth and organizational belonging

Socioemotional wealth (SEW) represents one of the central theoretical perspectives used to explain the distinctive behavior of family firms (Helland, 1988). SEW refers to the non-economic value that family owners derive from their involvement in the business, including family control and influence, identification of family members with the firm, emotional attachment, social ties, and the intention to preserve the family legacy across generations (Berrone et al., 2012; Kammerlander, 2022). Because these socioemotional endowments are closely connected to family identity and long-term continuity, family firms often prioritize the preservation of SEW alongside financial performance when making strategic decisions (Berrone et al., 2012; Rehman et al., 2023).

The pursuit of socioemotional wealth can shape the internal organizational environment of family firms by emphasizing relational values, trust, and long-term orientation (Eddleston and Jennings, 2024; Hayward et al., 2022). These characteristics frequently foster stronger interpersonal relationships and social cohesion within the organization, which may influence how employees perceive their connection to the firm (Herrero, 2018; Sanchez-Ruiz et al., 2019; Hengst et al., 2020). Prior studies have suggested that family firms often cultivate strong relational climates that support cooperation, commitment, and identification among employees (Neckebrouck et al., 2018). Such relational dynamics may contribute to employees’ perceptions of belonging within the organization.

Organizational belonging refers to the psychological experience of feeling accepted, valued, and integrated within a social or organizational group (Blau et al., 2023; Urrila et al., 2025). A strong sense of belonging emerges when individuals perceive that they are recognized members of the organization and that their contributions are appreciated by colleagues and leaders (Bryer, 2020; Davis et al., 2022; Xu and Luo, 2025). Research has consistently shown that belonging is associated with important positive outcomes, including greater organizational commitment, enhanced engagement, and improved collaboration among employees (Canlas and Williams, 2022; Klein et al., 2025). Conversely, when individuals experience exclusion or identity mismatch within the organization, their sense of belonging and attachment may decline (Kim et al., 2019; Piwowar-Sulej, 2021).

In family firms, socioemotional priorities may play an important role in shaping employees’ sense of belonging because these firms frequently emphasize shared identity, relational continuity, and collective values (Eddleston and Jennings, 2024; Casprini et al., 2024). The close interaction between family members and employees can create organizational environments where personal relationships, loyalty, and mutual commitment are particularly salient (Salvato et al., 2019; Herrero and Hughes, 2019; Zhang et al., 2025). Such conditions may strengthen employees’ perceptions that they are part of a cohesive organizational community rather than merely participants in an economic exchange relationship.

At the same time, socioemotional wealth may influence belonging through the development of social capital within the organization. Family firms often rely on dense relational networks and shared norms that facilitate cooperation and trust among organizational members (Carr et al., 2011; de Groot et al., 2022; Cisneros et al., 2022). Social capital can enhance communication, support knowledge sharing, and strengthen employees’ identification with the organization (Cai et al., 2020; Stasa and Machek, 2022). As a result, socioemotional priorities that promote relational closeness and shared identity may contribute to stronger feelings of belonging among employees.

Overall, the SEW perspective suggests that the socioemotional goals pursued by family owners may influence not only strategic decisions but also the relational environment experienced by employees. By emphasizing identity, continuity, and interpersonal relationships, socioemotional wealth may create organizational contexts in which employees experience stronger psychological attachment and belonging to the firm. Accordingly, examining the relationship between socioemotional wealth and employees’ organizational belonging provides an important step toward understanding the employee-level implications of family influence within organizations.

### The mediating role of family governance

Understanding how family-level orientations translate into individual experiences within family firms remains an important issue in family business research. In multigenerational family firms, the presence of strong family values or relational resources does not automatically ensure that individual family members experience a sense of inclusion within the organization. Institutional arrangements that structure family participation in the firm may therefore play an important role in shaping how broader family dynamics are reflected in organizational experiences. In this regard, family governance practices provide a useful lens for examining how family-level priorities and relationships are linked to individual-level outcomes.

Family governance refers to the formal and informal mechanisms that organize the relationship between the family and the business, including structures such as family councils, family assemblies, family constitutions, and regular family meetings (Barrett and Moores, 2020). These arrangements create structured settings in which family members communicate expectations, clarify roles, and coordinate decision-making. Beyond their administrative functions, governance practices also provide arenas where shared values and norms related to the family firm are discussed and reinforced. In multigenerational contexts, such mechanisms can be particularly relevant because increasing family size, generational diversity, and varying levels of involvement may complicate informal coordination among family members.

From the perspective of socioemotional wealth (SEW), families often seek to preserve non-economic values such as family influence, identity, and continuity across generations (Gómez-Mejía et al., 2007; Berrone et al., 2012). Governance practices may be associated with these priorities insofar as they provide mechanisms through which families organize participation in the business while maintaining shared values and collective identity. For example, family councils or constitutions may help articulate expectations about family involvement, succession, and communication, thereby helping families manage the complexities of multigenerational participation while protecting elements of socioemotional wealth.

At the same time, governance practices may shape how individual family members experience their relationship with the organization. A social identity perspective suggests that individuals derive part of their self-concept from membership in meaningful social groups (Ashforth and Mael, 1989). In family firms, governance forums can function as spaces where membership boundaries, shared narratives, and collective goals are articulated. Participation in these settings may reinforce individuals’ perception that they are recognized and included members of the organizational community. In this sense, governance practices may be associated with how family members experience organizational belonging, which reflects feelings of acceptance, inclusion, and connection within the workplace.

Importantly, family social capital characterized by trust, shared norms, and strong relational ties among family members may provide the relational foundation for these governance processes. However, relational resources alone may not fully explain how family members experience belonging within the organization, particularly in larger or more complex family firms. Governance practices may help translate relational dynamics into more visible and structured forms of interaction, thereby shaping the context in which family members engage with the organization.

Consistent with the cross-sectional design of the present study, family governance is therefore examined as a variable that may account for part of the observed association between family social capital and organizational belonging. Rather than implying a definitive causal pathway, this approach considers whether patterns in the data are consistent with a mediating relationship in which governance practices are associated with both family relational resources and individual experiences of belonging within the firm. This perspective provides a more nuanced understanding of how family-level dynamics, institutional arrangements, and individual psychological experiences are connected in multigenerational family firms.

### Theoretical model and hypotheses

**Figure 1:**
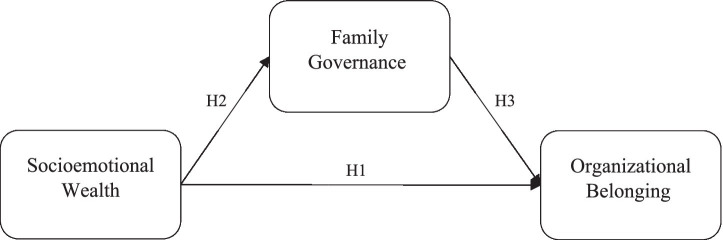
The conceptual model: a moderated framework.

Family firms are distinctive organizational contexts in which family relationships, governance arrangements, and organizational participation intersect. In multigenerational family firms, the interaction between family identity and organizational structures becomes particularly important for understanding how family members experience their roles within the firm. This study draws on a social identity perspective to examine the relationships among socioemotional wealth (SEW), family governance, and organizational belonging (Figure 1).

Socioemotional wealth refers to the non-economic value family members derive from their involvement in the firm (Gómez-Mejía et al., 2007; Berrone et al., 2012). The SEW framework emphasizes family-centered priorities such as the preservation of family influence, the maintenance of family identity in the business, emotional attachment to the firm, protection of the family’s reputation, and the continuity of family involvement across generations. These priorities often shape how families organize their participation in the business and how they structure governance arrangements. In multigenerational family firms, governance practices such as family councils, family assemblies, family constitutions, and structured communication forums serve as institutional mechanisms that help coordinate family participation and maintain shared expectations regarding the family–business relationship.

From this perspective, socioemotional wealth priorities may be associated with the adoption and strengthening of family governance practices. Families that place strong value on preserving family identity, influence, and continuity may be more likely to develop governance structures that facilitate coordination among family members and sustain shared family values within the firm. However, because family firms evolve over time and governance structures may also shape family relationships and perceptions, the relationship between SEW and governance practices should not be interpreted as strictly directional. Instead, the present study examines whether these constructs are empirically associated within multigenerational family firms.

*H1*: Socioemotional wealth is positively associated with family governance practices in multigenerational family firms.

At the individual level, family members’ experiences within the firm are reflected in their sense of organizational belonging, which refers to the extent to which individuals feel accepted, included, and connected within their organizational environment. According to social identity theory (Ashforth and Mael, 1989), individuals derive part of their self-concept from their membership in meaningful social groups. In family firms, the overlap between family and organizational membership may intensify these identity processes.

Family governance practices may be particularly relevant in this context because they create formal spaces for communication, participation, and collective decision-making among family members. Through mechanisms such as family meetings or councils, governance arrangements may reinforce shared narratives about the family’s role in the firm and clarify expectations regarding participation and responsibility. These processes may be associated with stronger perceptions of inclusion and recognition within the organization, thereby relating to family members’ experiences of organizational belonging.

*H2*: Family governance practices are positively associated with family members’ organizational belonging in multigenerational family firms.

Building on these relationships, family governance practices may also be relevant to the association between socioemotional wealth and organizational belonging. Families that emphasize socioemotional priorities such as maintaining family identity, strengthening family ties, and ensuring continuity across generations may be more likely to develop governance arrangements that institutionalize these values within the firm. In turn, these governance practices may be associated with family members’ perceptions of inclusion, recognition, and participation in the organization.

However, given the cross-sectional design of this study, mediation should be interpreted cautiously. The analysis does not establish causal mechanisms or temporal ordering among the variables. Instead, the study examines whether the observed pattern of statistical associations among socioemotional wealth, family governance, and organizational belonging is consistent with partial mediation.

*H3*: Family governance practices statistically account for part of the association between socioemotional wealth and family members’ organizational belonging.

## Methodology

### Sampling strategy

Data were collected through a structured web-based questionnaire administered to members of Chinese family firms between July and September 2024. The study employed a cross-sectional research design, capturing respondents’ perceptions at a single point in time. Accordingly, the findings are interpreted as associations among socioemotional wealth, family governance, and organizational belonging, rather than causal relationships.

Because comprehensive lists of family firm members are difficult to obtain, the study used a combination of convenience and snowball sampling. Initial participants were recruited through professional networks, alumni associations, and family business contacts, and were asked to share the survey with other family members affiliated with their firms. Although this approach facilitated access to family firm respondents, it represents a non-probability sampling strategy, which limits the generalizability of the findings. A family firm was defined as a business in which ownership and managerial influence are concentrated within a single family and where the family intends to maintain control across generations (Chua et al., 1999; Astrachan et al., 2002). Respondents were eligible if their family owned a controlling share of the business and at least one family member was actively involved in management or operations.

The final sample included 454 respondents affiliated with family-owned firms. Participants reported different levels of involvement in the business, including active managerial roles, ownership participation, or occasional involvement in family governance activities. While this diversity allows the study to capture a range of family member perspectives, the presence of less-active participants should be considered when interpreting the results. To focus on multigenerational family firms, respondents confirmed that more than one generation of the owning family had been involved in the firm. However, the study did not collect matched data from multiple generations within the same firm, meaning the analysis reflects individual perceptions of family members rather than direct comparisons across generations within firms. Table 1 summarizes the demographic and firm-related characteristics of the respondents.

**Table 1:** Sample characteristics (*N* = 454).

Characteristic	Category	Frequency (n)	Percentage (%)
Gender	Male	284	62.56
	Female	170	37.44
Age	Under 30 years	82	18.06
	30–39 years	126	27.75
	40–49 years	118	25.99
	50–59 years	84	18.50
	60 years and above	44	9.70
Education level	High school or below	96	21.15
	Bachelor’s degree	208	45.81
	Master’s degree	114	25.11
	Doctorate or equivalent	36	7.93
Role in the firm	Founder/owner	128	28.19
	Next-generation family member	184	40.53
	Non-family executive/manager	142	31.28
Generation in family	First generation	132	29.07
	Second generation	234	51.54
	Third generation or later	88	19.39
Firm age	Less than 20 years	96	21.15
	20–40 years	178	39.21
	41–60 years	112	24.67
	More than 60 years	68	14.98

Table 1 presents the demographic and organizational characteristics of the sample. The respondents consisted of 454 individuals involved in family firms. The majority of respondents were male (62.56%), while 37.44% were female. In terms of age distribution, most participants were between 30 and 49 years old. Regarding education, nearly half of the respondents held a bachelor’s degree, followed by master’s degrees and high school qualifications. With respect to organizational roles, 28.19% of respondents were founders or owners, 40.53% represented next-generation family members, and 31.28% were non-family executives or managers. In terms of family generation, more than half of the respondents belonged to the second generation, while smaller proportions were from the first generation and third generation or later. Finally, the firms represented in the sample varied in age, with the largest proportion operating between 20 and 40 years, followed by firms aged between 41 and 60 years. A smaller share of firms had been established for more than 60 years, reflecting the presence of long-standing multigenerational family businesses.

### Measurement and analysis

Table 2 presents the descriptive statistics and correlation matrix for the key variables included in this study. The results indicate that socioemotional wealth is positively associated with both family governance and organizational belonging. In particular, the correlation between socioemotional wealth and organizational belonging is relatively strong, suggesting that the preservation of family-centered non-economic values is closely related to individuals’ sense of belonging within the organization. Family governance also shows a positive association with organizational belonging, indicating that governance structures and family-related decision-making processes may contribute to strengthening members’ psychological connection to the firm. Regarding the control variables, firm age and generation in the family display moderate positive correlations with the main constructs. These findings suggest that more established firms and later-generation involvement may be associated with stronger family governance practices and a greater sense of organizational belonging. Overall, none of the correlations exceed commonly accepted thresholds, indicating that multicollinearity is unlikely to be a concern in the subsequent analyses (Baron and Kenny, 1986).

**Table 2:** Descriptive statistics and correlations.

Variable	Mean	SD	1	2	3	4	5
1. Socioemotional wealth	3.87	0.71	1				
2. Family governance	3.65	0.76	0.46**	1			
3. Organizational belonging	3.92	0.68	0.52**	0.48**	1		
4. Firm age	2.33	0.95	0.18*	0.21*	0.16*	1	
5. Generation in family	1.90	0.74	0.27**	0.24**	0.29**	0.33**	1

*p < 0.05, **p < 0.01.

Table 3 presents the measurement model results for the key constructs included in this study. Socioemotional wealth was modeled as a multidimensional construct based on the FIBER framework, encompassing 10 dimensions related to family control, identity, social ties, emotional attachment, and succession dynamics. In addition, family governance and organizational belonging were included as central constructs in the research model. The results indicate that all factor loadings are statistically significant and fall within acceptable ranges. Composite reliability values exceed the recommended threshold of 0.70, and the average variance extracted values are above 0.50, demonstrating satisfactory internal consistency and convergent validity. These findings confirm the adequacy of the measurement model and support the use of these constructs in the subsequent structural analysis (Podsakoff et al., 2003).

**Table 3:** Measurement model of study constructs.

Construct	Dimension	Factor loading range	CR	AVE	Cronbach’s α
Socioemotional wealth (FIBER)	Family control	0.74–0.86	0.88	0.55	0.83
	Family commitment	0.71–0.84	0.86	0.52	0.81
	Family identity	0.76–0.89	0.91	0.63	0.87
	Family reputation	0.75–0.88	0.90	0.61	0.86
	Social ties	0.69–0.83	0.85	0.51	0.80
	Family cohesion	0.73–0.85	0.88	0.56	0.84
	Emotional attachment	0.78–0.90	0.92	0.66	0.89
	Shared family values	0.74–0.87	0.89	0.58	0.85
	Succession renewal	0.70–0.82	0.86	0.53	0.82
	Family legacy	0.76–0.88	0.90	0.61	0.86
Family governance	Governance structure	0.72–0.85	0.89	0.58	0.84
	Family council practices	0.74–0.88	0.90	0.60	0.86
	Decision-making participation	0.71–0.84	0.87	0.55	0.82
Organizational belonging	Psychological belonging	0.77–0.90	0.91	0.63	0.88
	Organizational identification	0.75–0.88	0.90	0.61	0.86
	Emotional connection to firm	0.73–0.87	0.89	0.58	0.85

All standardized factor loadings are significant at *p* < 0.001.

Table 4 reports the discriminant validity for all measurement dimensions across socioemotional wealth, family governance, and organizational belonging. The results satisfy the Fornell–Larcker criterion, as the square roots of AVE (diagonal values) exceed all corresponding inter-construct correlations. Likewise, all HTMT ratios are below 0.85, confirming satisfactory discriminant validity (Schaufeli et al., 2006). These outcomes demonstrate that each of the 10 dimensions of socioemotional wealth, the three dimensions of family governance, and the three dimensions of organizational belonging are empirically distinguishable. The results further validate the multidimensional conceptualization of SEW and its theoretical distinctiveness from governance mechanisms and members’ organizational belonging.

**Table 4:** Discriminant validity of study constructs.

Construct	FC	FCom	FId	FRep	ST	Coh	EA	SFV	SR	FL	GS	FCP	DMP	PB	OI	EC	Max HTMT
FC	**0.74**	0.63	0.55	0.49	0.47	0.45	0.43	0.41	0.39	0.38	0.35	0.34	0.33	0.31	0.30	0.29	**0.71**
FCom		**0.72**	0.58	0.52	0.49	0.47	0.45	0.44	0.40	0.39	0.37	0.36	0.34	0.33	0.32	0.30	**0.71**
FID			**0.79**	0.63	0.59	0.54	0.51	0.49	0.46	0.45	0.42	0.40	0.38	0.36	0.35	0.33	**0.68**
FRen				**0.77**	0.61	0.57	0.53	0.51	0.47	0.44	0.41	0.39	0.37	0.36	0.35	0.34	**0.69**
ST					**0.71**	0.63	0.58	0.54	0.51	0.46	0.43	0.41	0.39	0.38	0.36	0.35	**0.69**
Coh						**0.75**	0.61	0.57	0.53	0.50	0.46	0.44	0.42	0.40	0.38	0.37	**0.65**
EA							**0.81**	0.66	0.59	0.54	0.49	0.47	0.44	0.43	0.41	0.40	**0.70**
SFV								**0.78**	0.61	0.57	0.52	0.48	0.45	0.44	0.42	0.41	**0.70**
SR									**0.75**	0.63	0.54	0.51	0.47	0.46	0.44	0.42	**0.66**
FL										**0.77**	0.52	0.49	0.45	0.44	0.42	0.41	**0.66**
GS											**0.75**	0.66	0.61	0.58	0.55	0.52	**0.70**
FCP												**0.76**	0.65	0.59	0.55	0.51	**0.69**
DMP													**0.74**	0.54	0.51	0.48	**0.65**
PB														**0.79**	0.67	0.63	**0.72**
OI															**0.78**	0.69	**0.73**
EC																**0.81**	**0.73**

FC, Family Control; Fcom, Family Commitment; FID, Family Identity; FRen, Family Reputation; ST, Social Ties; Coh, Family Cohesion; EA, Emotional Attachment; SFV, Shared Family Values; SR, Succession Renewal; FL, Family Legacy; GS, Governance Structure; FCP, Family Council Practices; DMP, Decision-Making Participation; PB, Psychological Belonging; OI, Organizational Identification; EC, Emotional Connection. Bold values on the diagonal represent the square root of the Average Variance Extracted (AVE) for each construct, demonstrating discriminant validity.

## Results

**Figure 2:**
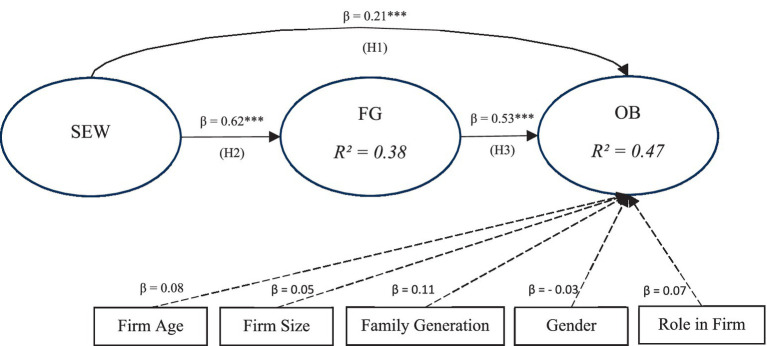
Structural model with standardized path coefficients. *** *p* < 0.001.

Figure 2 presents the structural model results for the relationships among socioemotional wealth (SEW), family governance (FG), and organizational belonging (OB). Socioemotional wealth is positively associated with organizational belonging (H1), and it is also positively associated with family governance (H2). In turn, family governance is positively associated with organizational belonging (H3). The indirect path from socioemotional wealth to organizational belonging through family governance is statistically significant, while the direct association between socioemotional wealth and organizational belonging remains significant after including family governance. These findings are consistent with a pattern of partial mediation, suggesting that family governance mechanisms are one, but not the only, pathway through which family-centered non-economic priorities relate to members’ sense of belonging in multigenerational family firms.

The results indicate that socioemotional wealth has a significant positive effect on family governance (*β* = 0.62, *p* < 0.001) and directly influences organizational belonging (*β* = 0.21, *p* < 0.01). In addition, family governance has a strong positive effect on organizational belonging (*β* = 0.53, *p* < 0.001). The model explains 38% of the variance in family governance (*R*^2^ = 0.38) and 47% of the variance in organizational belonging (*R*^2^ = 0.47). Several control variables firm age, firm size, family generation, gender, and role in the firm were also included, with paths directed toward organizational belonging. Standardized coefficients are reported. Significance levels are indicated as ***p < 0.001, **p < 0.01, *p < 0.05.

The structural model results provide strong support for the proposed theoretical relationships among socioemotional wealth (SEW), family governance (FG), and organizational belonging (OB). The findings indicate that SEW has a significant and positive effect on family governance (*β* = 0.62, *p* < 0.001), suggesting that higher levels of socioemotional wealth encourage the development of stronger and more structured governance mechanisms within family firms. In turn, family governance demonstrates a substantial positive effect on organizational belonging (*β* = 0.53, *p* < 0.001), indicating that effective governance practices contribute to a stronger sense of attachment and identification with the organization.

In addition to the indirect pathway, SEW also shows a direct positive effect on organizational belonging (*β* = 0.21, *p* < 0.01). This result suggests that socioemotional wealth influences employees’ sense of belonging both directly and indirectly through governance mechanisms. The indirect effect of SEW on OB through FG (*β* = 0.33) confirms the presence of partial mediation, indicating that family governance serves as an important mechanism through which socioemotional wealth enhances organizational belonging. The explanatory power of the model is satisfactory, with SEW accounting for 38% of the variance in family governance (*R*^2^ = 0.38) and the overall model explaining 47% of the variance in organizational belonging (*R*^2^ = 0.47). These values indicate moderate to substantial explanatory capability in the context of organizational and family business research. Furthermore, the inclusion of control variables firm age, firm size, family generation, gender, and role in the firm does not substantially alter the main relationships, although family generation shows a modest positive effect on organizational belonging. Overall, the results confirm the robustness of the proposed structural model and highlight the central role of family governance as a key mechanism linking socioemotional wealth to employees’ sense of organizational belonging in family firms.

### Mediation analysis

The bootstrapping results reported in Table 5 provide evidence regarding the mediating role of family governance (FG) in the relationship between socioemotional wealth (SEW) and organizational belonging (OB). First, the direct effect of SEW on OB is positive and statistically significant (*β* = 0.21, *p* < 0.01). This finding indicates that higher levels of socioemotional wealth within family firms are associated with a stronger sense of organizational belonging among employees. In other words, when family firms emphasize emotional attachment, family identity, and the preservation of family-centered values, employees tend to develop a stronger psychological connection to the organization. Second, SEW shows a strong and highly significant effect on family governance (*β* = 0.62, *p* < 0.001). This result suggests that firms with stronger socioemotional wealth orientations are more likely to establish structured governance mechanisms, such as clear decision-making processes, formal family involvement structures, and coordinated family-business relationships. These governance mechanisms help translate family values into organizational practices. Third, family governance has a significant positive effect on organizational belonging (*β* = 0.53, *p* < 0.001). This finding implies that well-structured governance systems contribute to employees’ sense of inclusion, identification, and attachment to the organization. Effective governance may create clearer communication channels, transparent decision-making processes, and stronger alignment between family and organizational values, which collectively reinforce employees’ sense of belonging.

**Table 5:** Mediation analysis (bootstrap results).

Path	Direct effect (β)	Indirect effect (β)	Total effect (β)	95% CI lower	95% CI upper	Mediation result
SEW → FG → OB	0.21**	0.33***	0.54***	0.19	0.45	Partial mediation (significant)
SEW → FG	0.62***	–	–	–	–	Significant
FG → OB	0.53***	–	–	–	–	Significant

***p < 0.001, **p < 0.01; Indirect/total effects and CIs are based on 5,000 bootstrap samples. Mediation result is based on significance of both direct and indirect paths.

Most importantly, the mediation analysis shows that the indirect effect of SEW on OB through FG is significant [*β* = 0.33, 95% CI (0.19, 0.45)]. Because the confidence interval does not include zero, the mediating effect is statistically supported. This indicates that a substantial portion of the influence of socioemotional wealth on organizational belonging operates through the development of family governance structures. The total effect of SEW on OB is relatively strong (*β* = 0.54, *p* < 0.001), reflecting the overall positive association between these variables. However, since both the direct effect (*β* = 0.21) and the indirect effect (*β* = 0.33) remain significant, the results support a partial mediation pattern. This suggests that family governance functions as an important mechanism through which socioemotional wealth enhances employees’ sense of belonging, while SEW also maintains a direct influence on belonging beyond governance structures. Taken together, these findings highlight the central role of governance mechanisms in translating the socioemotional priorities of family firms into meaningful organizational outcomes. Specifically, socioemotional wealth not only directly strengthens employees’ attachment to the organization but also indirectly reinforces belonging by encouraging the development of governance structures that institutionalize family values within the firm.

### Multi-group analysis by gender

To examine whether the structural paths differed across demographic subgroups, a multi-group analysis (MGA) was conducted in AMOS using gender as the grouping variable. Prior to comparing structural paths, measurement invariance was established across male and female respondents. Configural, metric, and scalar invariance tests indicated acceptable fit (ΔCFI < 0.01; ΔRMSEA < 0.015), supporting valid comparability. Two structural models were then estimated separately for male (n₁ = 228) and female (n₂ = 226) participants. Table 6 presents the standardized coefficients.

**Table 6:** Multi-group analysis results by gender.

Path	Male (β)	Female (β)	Δβ	χ^2^-diff (p)	Moderation result
SEW → FG	0.65***	0.59***	0.06	1.87 (ns)	Not significantly different
FG → OB	0.47***	0.58***	−0.11	4.52*	Significant difference
SEW → OB (direct)	0.25**	0.18*	0.07	2.21 (ns)	Marginal difference

***p < 0.001, **p < 0.01, *p < 0.05; ns, not significant. Critical ratio differences > ± 1.96 indicate significant moderation.

The gender-based differences observed in the MGA provide exploratory indications of how employees may perceive governance arrangements and socioemotional priorities differently. The slightly stronger SEW → FG path among male respondents might be interpreted as reflecting differential exposure to or familiarity with governance processes in family firms. In many traditional contexts, men tend to hold more central roles in strategic and ownership-related activities (Miller and Le Breton-Miller, 2006). Such roles could be consistent with a more direct alignment between socioemotional priorities and governance perceptions. However, these interpretations are speculative, as the mechanisms underlying these differences (e.g., role expectations or involvement levels) were not directly measured or tested in this study.

In contrast, the stronger FG → OB association among female respondents may suggest that transparent and structured governance arrangements resonate more strongly with women’s perceptions of relational clarity and procedural fairness. Prior work has noted that women in family firms often emphasize inclusive communication and fairness-oriented interactions (Eddleston and Kidwell, 2012). Nevertheless, the present data do not allow causal conclusions, and these potential explanations remain tentative and are offered only as possible interpretations.

### Multi-group analysis by age

To further examine whether structural relationships varied across demographic segments, a multi-group analysis (MGA) was conducted in AMOS using age as the grouping variable. Respondents were divided into two groups based on the sample distribution: younger employees (≤35 years) and older employees (>35 years). Prior to comparing structural paths, measurement invariance across the two age groups was assessed. The results supported configural and metric invariance (ΔCFI < 0.01), indicating that the measurement model functioned equivalently across age groups and allowing meaningful comparison of structural relationships. Separate structural models were then estimated for the two age groups. The standardized path coefficients for each group are presented in Table 7.

**Table 7:** Multi-group analysis by age.

Path	Younger (≤35) β	Older (>35) *β*	Δβ	χ^2^-diff (p)	Moderation result
SEW → FG	0.58***	0.66***	−0.08	2.03 (ns)	Not significantly different
FG → OB	0.49***	0.56***	−0.07	3.91*	Significant difference
SEW → OB	0.24**	0.19*	0.05	1.72 (ns)	Not significantly different

*p < 0.05, **p < 0.01, ***p < 0.001; ns, not significant.

The MGA results indicate some variation in the strength of structural associations across age groups. The SEW → OB relationship appears somewhat stronger among younger respondents, whereas the FG → OB association is relatively stronger among older participants. These differences should be interpreted as descriptive patterns rather than causal effects. One possible interpretation is that employees in earlier career stages may be particularly attentive to socioemotional signals within family firms, such as family identity, trust, and symbolic recognition (Zellweger et al., 2012). Such cues may be associated with how younger individuals perceive their psychological connection to the organization, especially when they are in stages of professional identity development or early organizational integration. Similarly, involvement in succession preparation processes might make socioemotional cues more salient for younger members of family firms (Sieger et al., 2011). However, these explanations remain speculative, as variables capturing identity formation or succession involvement were not directly measured in the present study.

For older respondents, the relatively stronger FG → OB association may suggest that governance clarity becomes increasingly relevant as employees accumulate experience and occupy more established organizational roles. Formalized governance mechanisms such as defined decision procedures, accountability structures, and clearer role expectations could be consistent with stronger perceptions of organizational stability and procedural transparency (Miller and Le Breton-Miller, 2014). Nevertheless, the current data do not allow firm conclusions about the underlying mechanisms, and these interpretations should be considered tentative. Overall, the age-based MGA findings suggest that demographic characteristics may be associated with differences in how employees relate socioemotional priorities and governance arrangements to their sense of organizational belonging. Given the cross-sectional design and the absence of direct measures of the proposed explanatory mechanisms, this subgroup differences should be viewed as exploratory insights that warrant further investigation in future research (Table 8).

**Table 8:** Dimension-wise mediation analysis results.

SEW dimension	Direct effect (SEW → OB)	Indirect effect (SEW → FG → OB)	Total effect	95% CI lower	95% CI upper	Mediation type
Emotional attachment (EA)	0.28	0.25	0.53	0.14	0.37	Partial
Family control and influence (FCI)	0.12	0.31	0.43	0.20	0.41	Full
Identification (IFM)	0.15	0.34	0.49	0.22	0.46	Full
Binding social ties (BST)	0.09	0.26	0.35	0.18	0.39	Full
Renewal of family bonds (RFB)	0.11	0.22	0.33	0.10	0.35	Partial
Family reputation (FR)	0.20	0.18	0.38	0.09	0.28	Partial
Transgenerational continuity (TC)	0.05	0.29	0.34	0.19	0.40	Full
Loyalty (LY)	0.23	0.20	0.43	0.11	0.34	Partial
Family harmony (FH)	0.08	0.25	0.33	0.15	0.38	Full
Shared vision (SV)	0.18	0.27	0.45	0.17	0.37	Partial

### Dimension-wise mediation analysis

The dimension-wise mediation analysis provides fine-grained evidence on how socioemotional wealth (SEW) is transformed into employees’ organizational belonging (OB) through family governance (FG). This approach directly responds to recent theoretical calls to “unpack the multidimensional nature of SEW rather than treating it as a global latent construct” (Berrone et al., 2012; Hauck et al., 2016).

The results of our analysis reveal heterogeneous mediation patterns across the 10 SEW dimensions, demonstrating that different socioemotional motives rely on different mechanisms to influence employee-level outcomes. First, five dimensions Family Control and Influence (FCI), Identification of Family Members with the Firm (IFM), Binding Social Ties (BST), Transgenerational Continuity (TC), and Family Harmony (FH) exhibit full mediation through FG. This finding aligns with the conceptualization that governance structures serve as the organizational translation mechanism for SEW-related motives (Miller and Le Breton-Miller, 2006; Chrisman et al., 2012). In particular, dimensions rooted in identity reinforcement (IFM, TC) and relational cohesion (BST, FH) appear highly dependent on structured governance systems (e.g., family councils, reporting rules, communication protocols) to shape employees’ sense of belonging. These results resonate with arguments by Zellweger et al. (2013), who emphasize that when SEW elements are deeply embedded in firm identity and family relational systems, their effects are most effectively transmitted via formal governance channels. Second, the remaining dimensions Emotional Attachment (EA), Renewal of Family Bonds (RFB), Family Reputation (FR), Loyalty (LY), and Shared Vision (SV) show partial mediation, indicating that their influence on OB is driven both by direct affective pathways and by indirect structural pathways. This dual mechanism supports the conceptual distinction between the affective and instrumental components of SEW (Gómez-Mejía et al., 2007; Debicki et al., 2016). Emotional attachment and shared vision, for example, create immediate psychological bonds (direct effect), but are further reinforced when governance structures institutionalize family values and expectations (indirect effect). Similarly, the mediation patterns observed for RFB and LY align with literature suggesting that intergenerational continuity and loyalty function simultaneously as interpersonal dynamics and as governance-driven norms (Jaskiewicz and Dyer, 2017).

Importantly, the observed mediation structure aligns with recent calls in family business research to position family governance as the central mechanism linking SEW to organizational outcomes (Daspit et al., 2023; Suess, 2014). Our findings empirically affirm this role: even in dimensions where direct effects remain significant, FG provides a consistent, routinized, and legitimized channel through which SEW manifests in employee-level perceptions of belonging. Furthermore, the mediation patterns correspond with the MGA results across gender and age. Dimensions characterized by relational cohesion (BST, FH, TC) show stronger indirect effects among women and older employees, consistent with identity-based and life-course theories (Eddleston and Powell, 2012; Sharma and Irving, 2005). This suggests that these demographic groups respond more strongly to structured expressions of SEW particularly those related to harmony, continuity, and relational bonds when codified through governance practices. Such alignment reinforces the robustness of our mediation findings and provides a multi-layered explanation integrating SEW theory, governance mechanisms, and employee identity dynamics. Overall, the results demonstrate that family governance is not merely an administrative layer but rather the primary transmission channel through which socioemotional wealth permeates organizational life and ultimately enhances employees’ organizational belonging. By systematically revealing which SEW dimensions rely on governance and which also exert independent emotional influence, this study advances a more nuanced understanding of both SEW and governance in family firms.

## Discussion

This study examined the associations between socioemotional wealth (SEW), family governance (FG), and employees’ organizational belonging (OB). The findings indicate that SEW is positively associated with both family governance and organizational belonging, while family governance also demonstrates a positive relationship with employees’ sense of belonging. The mediation analysis further suggests that family governance is consistent with an indirect path through which socioemotional priorities are associated with employees’ psychological attachment to the organization. These results provide an empirical validation of how socioemotional priorities are reflected in organizational processes within the specific context of Chinese family firms.

Previous research has emphasized that family firms often prioritize non-economic goals, such as family control, identity, and reputation (Gómez-Mejía et al., 2007; Berrone et al., 2012). While these socioemotional priorities are often linked to strategic and governance choices, the present findings suggest that governance arrangements may represent one of the organizational channels associated with how these priorities are perceived by employees. In this respect, family governance structures such as formalized decision procedures or family councils may help institutionalize family values by aligning them with organizational practices (Gersick et al., 1997; Suess, 2014). When governance arrangements provide clarity regarding roles and expectations, this clarity is associated with greater perceived procedural fairness, which in turn relates to a stronger sense of belonging. This interpretation is consistent with literature suggesting that governance structures in family firms may reduce ambiguity and are associated with organizational legitimacy (Miller and Le Breton-Miller, 2006).

Importantly, as this study employs a cross-sectional design, these results should be interpreted as associational rather than strictly causal. Rather than suggesting that socioemotional wealth directly produces stronger belonging, the findings indicate that the presence of governance mechanisms is associated with a stronger link between family values and employees’ perceptions. In this sense, governance may be viewed as an institutional bridge through which socioemotional priorities are expressed within the organizational context. The observed indirect relationship also highlights the potentially complementary roles of values and structures. Socioemotional wealth may be related to employees’ perceptions through shared identity and the symbolic presence of the family (Zellweger et al., 2012), while governance practices may reinforce these associations by embedding family priorities into everyday organizational processes. This suggests that both relational and structural dimensions of family involvement are relevant for understanding employees’ organizational attachment.

The multi-group analyses (MGA) provide exploratory insights into how governance signals may be interpreted differently across employee groups. The stronger association between family governance and organizational belonging observed among women might suggest a potential sensitivity to relational stability and transparent communication; however, this interpretation remains speculative as these underlying psychological mechanisms were not directly tested in the current study. Similarly, the stronger relationship observed among older employees may be interpreted in light of the possibility that individuals with greater organizational experience place higher value on structured governance. While governance clarity might reinforce perceptions of stability elements often associated with the long-term orientation of family firms (Le Breton-Miller and Miller, 2016) these demographic differences are tentative and require further validation in future research specifically designed to test these underlying motivations.

In conclusion, these findings suggest that family governance is meaningfully associated with the way socioemotional priorities are reflected in organizational experiences. Rather than being framed as a causal driver, governance appears to act as a contextual bridge connecting family-centered values with employees reported psychological attachment. By providing structure and procedural transparency, governance mechanisms are associated with organizational practices that correlate with a stronger sense of belonging among employees. These results offer a contextual extension of SEW theory by applying its arguments to employee-level outcomes in a non-Western setting.

## Theoretical implications

This study offers several focused and measured theoretical contributions to the literature on family firms, socioemotional wealth (SEW), and family governance (FG), specifically providing a contextual extension of these frameworks to employee-level outcomes. First, the study provides empirical validation of SEW theory by examining how socioemotional priorities are associated with employees’ organizational experiences through structural mechanisms. While SEW research has traditionally focused on strategic behavior and risk preferences at the family or top management level (Gómez-Mejía et al., 2007; Berrone et al., 2012; Buachoom et al., 2023), the pathways through which socioemotional values relate to micro-level employee outcomes have remained less explored. By observing that family governance is consistent with an indirect path between SEW and organizational belonging, this study suggests that SEW priorities may be reflected not only symbolically but also institutionally, through organizational structures that communicate family values to the broader workforce.

Second, the findings extend the literature on family governance by considering governance as more than a control or coordination device (Gersick et al., 1997; Suess, 2014; Tian et al., 2025). The results are consistent with the view that when governance practices clarify procedures and formalize communication, they may serve as a structural bridge through which socioemotional motives are associated with employees’ perceptions of stability and legitimacy. This offers a contextual application of governance theory, reinforcing perspectives that view governance as possessing both procedural and symbolic dimensions (Miller and Le Breton-Miller, 2006; Frick et al., 2025) within the specific institutional setting of Chinese family firms.

Third, the study offers exploratory insights into the micro-foundations of family firms by identifying variations in structural associations across demographic groups, such as gender and age. While prior work acknowledges that SEW may be perceived differently depending on an individual’s proximity to the family (Zellweger et al., 2012; Sorenson and Milbrandt, 2023), less is known about how demographic characteristics relate to the interpretation of governance signals. Our multi-group findings suggest that the associations between governance clarity and belonging may be more pronounced among women and older employees. However, these findings are exploratory and speculative, as the specific psychological or relational mechanisms underlying these differences such as role expectations or identity formation were not directly tested in this study. This contribution highlights the importance of a more nuanced and demographic-sensitive lens in future SEW research.

Finally, the study contributes by highlighting how family-centered values and organizational structures are jointly associated with employee experiences. Rather than treating SEW as an abstract orientation or governance as a purely formal system, the results point toward their potentially complementary nature. This integrative perspective offers a grounded and context-sensitive application of SEW theory, identifying how socioemotional priorities may be reflected in the sense of belonging among employees. Collectively, these contributions do not claim to advance new theoretical laws but rather provide empirical support and contextual refinement for existing theories within the under-researched area of employee-level outcomes in family-owned enterprises.

## Practical implications

The findings of this study offer several practical implications for family business owners, managers, and governance designers seeking to strengthen employees’ organizational belonging. First, the results suggest that well-structured family governance mechanisms can enhance employees’ sense of belonging. Practices such as formal family councils, clarified decision-making processes, transparent communication channels, and explicit governance policies can help institutionalize family values and reduce ambiguity for employees. By integrating socioemotional priorities such as fairness, long-term orientation, and relational harmony into governance structures, family firms can create work environments that feel stable and trustworthy for organizational members.

Second, the findings imply that communicating socioemotional intentions clearly matters. Employees are more likely to build attachment when they understand how family values influence organizational practices. Managers can therefore reinforce belonging by making family priorities visible for example, through internal communication, leadership behavior that reflects family norms, or involvement of employees in discussions about organizational purpose and long-term vision.

Third, the demographic differences observed in the multi-group analysis highlight the importance of tailored governance communication. Women, who in many workplace contexts value relational consistency and procedural clarity, appear to be particularly responsive to governance structures that signal fairness and inclusiveness. Similarly, older employees may derive a stronger sense of belonging from governance arrangements that provide continuity, role clarity, and predictable organizational processes. Family firms may benefit from designing governance systems that account for such heterogeneous expectations.

Finally, the results emphasize that family governance should be viewed not only as an administrative requirement but also as a strategic tool for cultural alignment. By aligning governance mechanisms with socioemotional priorities, family firms can foster organizational environments where employees feel connected to the firm’s values and identity. This alignment can support higher engagement, stronger relational cohesion, and more stable employment relationships over time.

## Limitations and future research

This study should be interpreted in light of several limitations that also open avenues for future research. First, the study relies on a cross-sectional research design, which limits the ability to draw causal conclusions about the relationships among socioemotional wealth (SEW), family governance (FG), and organizational belonging (OB). Although the structural model identifies significant associations consistent with theoretical expectations, the temporal ordering of these relationships cannot be definitively established. Future studies could address this limitation by employing longitudinal or panel designs that examine how changes in governance structures or socioemotional priorities influence employee perceptions over time.

Second, the data were collected through self-reported survey responses, which may introduce the risk of common method variance. While statistical procedures were applied to minimize this concern, perceptions of governance practices and organizational belonging were captured from the same respondents. Future research could strengthen validity by using multi-source data, for example combining employee perceptions with managerial reports or archival governance information.

Third, the study focuses on a specific national and organizational context, which may limit the generalizability of the findings to other institutional environments. Governance structures, family norms, and employee expectations can vary substantially across cultural and regulatory settings. Comparative studies across countries or institutional contexts would help clarify how broader environments shape the relationship between SEW, governance practices, and employee outcomes.

Fourth, the analysis concentrates on family governance as a single mediating mechanism, while other organizational processes may also translate socioemotional priorities into employee experiences. Future research could explore additional mediators such as leadership style, organizational justice perceptions, internal communication practices, or psychological safety to better understand the micro-level mechanisms linking family influence to employee attitudes.

Finally, the multi-group analysis revealed differences across gender and age groups, suggesting that employee characteristics may condition how governance signals are interpreted. Future studies could further investigate these contingencies by examining other forms of heterogeneity, such as tenure, family versus non-family employee status, hierarchical position, or generational stage within the family firm. Addressing these limitations would deepen understanding of how socioemotional priorities are translated into organizational outcomes and would help develop a more comprehensive micro foundational perspective on governance and employee experience in family firms.

## Conclusion

This study examined how socioemotional wealth (SEW) relates to employees’ organizational belonging (OB) in family firms and whether family governance (FG) serves as a mechanism linking these constructs. Drawing on socioemotional wealth theory and governance perspectives, the study proposed that family governance structures help translate family-centered values into organizational practices that shape employees’ experiences within the firm. The findings indicate that socioemotional wealth is positively associated with both family governance and employees’ sense of organizational belonging. In addition, the mediation analysis shows that family governance partially mediates the relationship between SEW and organizational belonging, suggesting that governance structures represent an important channel through which socioemotional priorities become visible and meaningful to employees. These results highlight that governance mechanisms may play a role not only in coordinating family and business interests but also in shaping the relational environment experienced by organizational members.

The multi-group analysis further suggests that the strength of some relationships varies across employee characteristics. In particular, the effect of family governance on organizational belonging appears stronger among women and older employees, indicating that governance clarity and procedural transparency may be interpreted differently across demographic groups. These findings point to the importance of considering employee heterogeneity when examining how governance structures influence organizational attitudes. Overall, the study contributes to a more integrated understanding of how family values, governance mechanisms, and employee perceptions interact within family firms. By highlighting the mediating role of family governance and the contingent nature of employee responses, the findings provide a basis for further research exploring the micro-level processes through which family influence shapes organizational outcomes.

## Data Availability

The datasets presented in this study can be found in online repositories. The names of the repository/repositories and accession number(s) can be found in the article/Supplementary material.
